# A Systematic Profiling of the Components of Kukeya Tablets, a Traditional Ethnic Medicine Prescription, by Ultra-High-Performance Liquid Chromatography–Quadrupole/Orbitrap High-Resolution Mass Spectrometry

**DOI:** 10.3390/ph18040457

**Published:** 2025-03-24

**Authors:** Gulimire Kahaer, Muhebaiti Muhetaer, Rahima Abdulla, Tao Wu, Yuqin Luo, Haji Akber Aisa

**Affiliations:** 1 State Key Laboratory Basis of Xinjiang Indigenous Medicinal Plants Resource Utilization, Xinjiang Technical Institute of Physics and Chemistry, Chinese Academy of Sciences, Urumqi 830011, China; gulimire0110@sina.com (G.K.); muhabbat@ms.xjb.ac.cn (M.M.); rahima@ms.xjb.ac.cn (R.A.); wutao@ms.xjb.ac.cn (T.W.); luoyq@ms.xjb.ac.cn (Y.L.); 2 University of Chinese Academy of Sciences, No. 19 (A) Yuquan Road, Shijingshan District, Beijing 100049, China

**Keywords:** chemical components, qualitative analysis, Kukeya tablet, UHPLC-Q-Orbitrap-HRMS

## Abstract

**Background:** Kukeya tablets (KYs), a traditional ethnic medicine prescription, are widely used to treat migraines and eye ailments in China. Despite their extensive clinical use, current knowledge on their therapeutic material basis is limited to a few major compounds, whereas certain minor ones have rarely been investigated. This study was conducted to screen and characterize the chemical components of KYs. **Methods:** A rapid and effective UHPLC-Q-Orbitrap-HRMS method was established. A mass spectrometry qualitative analysis strategy for KYs was developed, including in-house library matching, accurate molecular mass and elemental composition matching, and MS/MS fragmentation rule elucidation. **Results:** In total, 144 compounds were identified in KYs, including 36 anthrones and anthraquinones, 36 chromones, 25 triterpenes, 12 resin glycosides, 12 phenylpyrones, 10 phenolic acids, 4 flavonoids, 2 lignans, and 7 others. Meanwhile, the identified compounds were effectively classified into nine chemical classes. Among them, 11 compounds were identified for the first time and the identities of 22 compounds were accurately confirmed using reference substances. **Conclusions:** The results obtained benefit the understanding of the therapeutic basis of KYs, significantly promote the quality control of KYs, and elucidate potential effective components of other traditional medicines.

## 1. Introduction

Kukeya tablets (KYs), an ethnic medicine prescription, are officially recognized by the Drug Specifications for the Ministry of Health of the People’s Republic of China, Uyghur drug volume (WS_3_-BW-0202-98), and are prepared from five medicinal materials, namely *Aloea barbadenis* Miller, *Artemisia absinthium* L., *Pistacia lentiscus* L., *Convolvulus scammonia* L., and *Citrullus colocynthis* (L.) Schard. Clinically, they have been used as the main prescription for treating migraine and eye ailments for several decades in China [1,2]. However, their chemical composition has only been studied for a single herb, specifically for anthrones, anthraquinones, chromones, and phenyl pyrones [3,4], which were isolated from *Aloea barbadenis* Miller and are the main phenolic metabolites present in aloe species, possessing various biological activities [4,5,6,7]. *Citrullus colocynthis* (L.) Schard., commonly known as “bitter apple” or “bitter cucumber”, is a plant which has been used for constipation, bacterial infections, edema, cancer, and diabetes [8,9,10,11,12]. Studies have previously reported that about 128 chemical compounds [11,12,13,14,15,16,17,18], including cucurbitacin-type triterpenoids, flavonoids, phenolic acids, alkaloids, sterols, coumarins, aromatics, fatty acids, and amino acids, have been isolated from *Citrullus colocynthis* (L.) Schard. Resin glycosides, which are characteristic of *Convolvulaceous* plants, are well known for their purgative properties [19]. Recently, there has been a renewed interest in these compounds due to the discovery of their novel biological activities, including cytotoxic effects on cancer cells, antibacterial and antiviral properties, anti-inflammatory actions, and the ability to modulate multidrug resistance [20,21,22,23,24]. Based on our previous study [25], a total of 80 compounds, including 58 resin glycosides and 22 glycosidic acids, were tentatively identified in *Convolvulus scammonia* L. *Artemisia absinthium* L. (wormwood) is a perennial herb, belonging to the *Artemisia* genus within the *Compositae* family. This plant is traditionally used for the treatment of parasites, anorexia, and indigestion [26,27]. There are existing studies which have reported [28,29,30] more than 80 compounds isolated from *Artemisia absinthium* L., mainly including sesquiterpenes, lignans, flavonoids, and phenolic acids. *Pistacia lentiscus* L. (mastic) [31] is a natural resin which is widely distributed in the dry and rocky regions of the Mediterranean. Pharmacological investigations of mastic have indicated that [31,32,33,34] it has anti-inflammatory, antioxidant, antibacterial, anticancer, and cardioprotective properties. Our previous studies showed that [35,36,37] about 59 compounds, including tirucallane-type tetracyclic triterpenes, olean, moronic, amyrone, lupane-type pentacyclic triterpenes, and pinane-type monoterpenes, were successfully isolated from mastic. Every herbal plant has its bioactivities, but their effects in a preparation are the comprehensive embodiment of all the active chemical components in a drug, characterized by multiple components, multiple targets, and their overall regulation. Therefore, it is necessary to develop a method to characterize the overall chemical profile of KYs, especially for their unknown and complicated constituents [38,39].

Lately, with the development of analytical testing technology, ultra-high-performance liquid chromatography combined with quadrupole/Orbitrap high-resolution mass spectrometry (UHPLC-HRMS) has been extensively applied for the detection and identification [38,39,40,41,42,43] of chemical constituents in Chinese traditional medicine (TCM). For example, some research groups successfully identified and quantified a variety of polyphenol compounds in several herb medicines. This not only offered valuable insights into the chemical composition of these herbs but also further emphasized the high sensitivity of UHPLC-MS methods [43,44,45]. Another research group, who developed a novel approach employing UHPLC-MS, allowed for the simultaneous detection and quantification of diverse bioactive components, including alkaloids, terpenoids, and organic acids. Through this study, it was demonstrated that UHPLC-MS could effectively separate and identify complex mixtures of bioactive compounds, even when faced with structural similarities among them [46,47].

In this study, the chemical constituents of KYs were systematically characterized by UHPLC-Q-Orbitrap-HRMS for the first time. Based on high-resolution MS data, combined with characteristic fragment ions (CFIs), predicted MS/MS fragmentation rules were used to identify complex chemical components. The results successfully revealed the complex and diverse chemical composition system of KYs, which will provide a data basis for future pharmacological investigations and the establishment of a safer and more effective and controllable quality control system.

## 2. Results and Discussion

**Figure 1:**
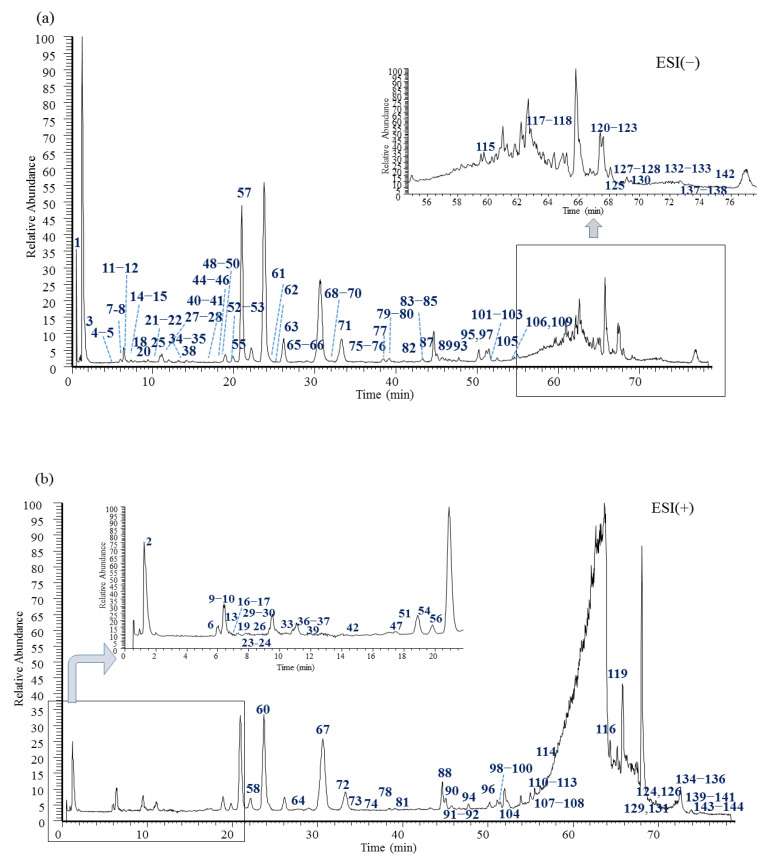
Total ion chromatogram of 70% methanol extracts of Kukeya tablets ((**a**): negative ion mode; (**b**): positive ion mode).

This study established a rapid and effective method using a UHPLC-Q-Orbitrap-HRMS system and developed mass spectrometric qualitative analysis strategies for KYs, including in-house library matching, accurate molecular mass and elemental composition matching, and MS/MS fragmentation rule elucidation. In total, 144 compounds, including 36 anthrones and anthraquinones, 36 chromones, 25 triterpenes—which were obtained from tetracyclic (including 9 tirucallane and 6 cucurbitacin types) and pentacyclic (including 7 olean and 3 lupane types) triterpenes—12 resin glycosides, 12 phenyl pyrones, 10 phenolic acids, 4 flavonoids, 2 lignans, and 7 others, were putatively identified. The MS data (MS^1^ and MS^2^), retention times, errors, identification, and classification of the above compounds are shown in Table S1 (in the Supplementary Materials). Among them, the identities of 15 compounds (**3**–**6**, **32**, **44**, **56**–**57**, **59**–**60**, **62**, **67**, **72**, **78**, and **103**) were confirmed through a comparison to reference substances. Moreover, the identities of seven compounds (**119**, **127**, **129**, **131**, **134**, and **139**–**140**) were assumed through a comparison with the compounds isolated in-house [37], which were from mastic, *Pistacia lentiscus* L. The total ion chromatogram (TIC) of the extracts of KYs is presented in Figure 1.

### 2.1. Identification of Anthrones and Anthraquinones

**Figure 2:**
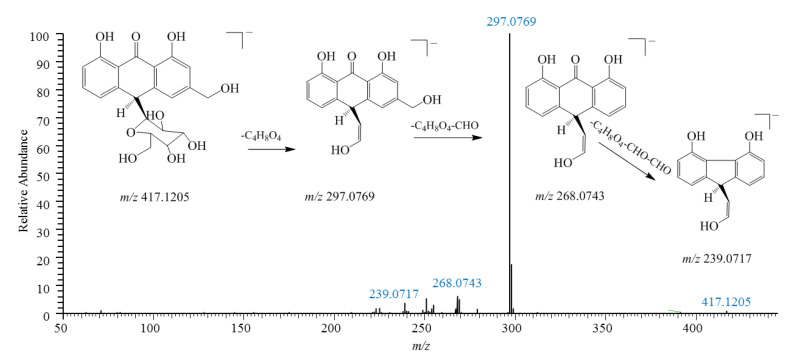
MS/MS spectra and proposed fragmentation pathways of aloin B (**57**) from *Aloea barbadenis* Miller, which was identified in KY.

In total, 36 anthrones and anthraquinones (**25**–**28**, **34**, **37**–**38**, **40**–**41**, **45**–**46**, **48**–**49**, **52**, **57**, **60**–**61**, **63**, **65**–**66**, **69**–**70**, **77**, **83**–**87**, **93**, **95**, **100**–**102**, **105**, and **108**–**109**) were detected in KY and were primarily derived from the source plant *Aloea barbadenis* Miller. In negative ion mode, anthrone/anthraquinone and their glycosides are prone to losing glucose units (C_4_H_8_O_4_) and CHO molecules [38]. For example, in a comparison with a reference substance, aloin B (**57**) exhibited [M-H]^−^ at *m*/*z* 417.1192 with a molecular formula of C_21_H_21_O_9_ and displayed fragment ions at *m*/*z* 297.0766 ([M-H-C_4_H_8_O_4_]^−^), *m*/*z* 268.0743 ([297-CHO]^−^), and *m*/*z* 239.0704 ([297-2CHO]^−^) in the MS^2^ spectrum, which were considered to be its CFIs (Figure 2). In addition, in positive ion mode, aloin A (**60**) displayed CFIs of *m*/*z* 257.0810 ([M+H-C_6_H_10_O_5_]^+^), *m*/*z* 239.0704 ([257-H_2_O]^+^), and *m*/*z* 211.0755 ([239-CO]^+^) in the MS/MS spectrum through the loss of a glucose unit and neutral molecules.

Compound (**28**) showed an adduct ion, [M-H+HCOO]^−^, at *m*/*z* 625.1791 with a molecular formula of C_28_H_33_O_16_, and its main fragments were obtained at *m*/*z* 417.1200 ([M-H+HCOO-C_6_H_10_O_5_]^−^) and *m*/*z* 255.0664 ([417-C_6_H_10_O_5_]^−^) in the MS^2^ spectrum; compared to aloin A/B, it was tentatively assumed to be aloin-O-hexoside, and compound **26** was its isomer. In addition, three compounds (**69**, **77**, and **86**) were identified as aloin derivatives, which also possessed main fragments of aloin; to the best of our knowledge, the above five compounds were identified for the first time in *Aloea barbadenis* Miller.

Regarding the anthraquinones, aloe emodin (**109**) had [M-H]^−^ at *m*/*z* 269.0402 with a molecular formula of C_15_H_19_O_5_. CFIs at *m*/*z* 240.0426 ([M-H-CHO]^−^) and *m*/*z* 211.0398 ([M-H-2CHO]^−^) were observed in the MS/MS spectrum.

Compound (**63**) produced an adduct ion, [M-H+HCOO]^−^, at *m*/*z* 609.1840 with a molecular formula of C_28_H_33_O_15_ and [M-H]^−^ at *m*/*z* 563.1776 with a molecular formula of C_27_H_31_O_13_, with its main fragment ions including *m*/*z* 443.1349 ([M-H-C_4_H_8_O_4_]^−^), *m*/*z* 295.0602 ([443-C_6_H_12_O_4_]^−^), and *m*/*z* 251.0712 ([295-CO_2_]^−^) in the MS/MS spectrum, produced through the successful loss of glucose groups and CO_2_ molecules; through a comparison with the literature [48], compound (**63**) was tentatively identified as aloinside B. In addition, three compounds (**65**, **85**, and **87**) that were isomers and derivatives of aloinside B were also detected, which were obtained from above the main fragments in their MS^2^ spectrum.

Compound (**102**) had [M-H]^−^ at *m*/*z* 709.2143 with a molecular formula of C_36_H_37_O_15_ and a highly abundant fragment at *m*/*z* 443.1145 ([M-H-C_4_H_8_O_4_-C_9_H_6_O_2_]^−^) in the MS^2^ spectrum, suggesting that glucosyl and coumaroyl groups existed. Comparing it with the literature [49,50,51], it was tentatively identified as microdontin-O-hexoside, which was identified for the first time in *Aloea barbadenis* Miller.

Based on the CFIs and proposed fragmentation pathways of the above compounds and data base searches (Compound Discoverer/Chem Blink/Chem Spider), a large number of compounds were identified as anthrones and anthraquinones [6,7,43,49,50,51]: aloin isomers/derivatives (**27**, **37**, and **61**), aloin-O-hexoside (**26**), 10-hydroxy-aloin B/A (**34** and **38**) and its isomers/derivatives (**25**, **45**, **52**, and **55**), aloe emodin-11-O-rhamnoside (**70**), 6′-O-acetyl-aloin A and its isomers/derivatives (**40**–**41**, **46**, **48**–**49**, **83**–**84**, and **93**), homonataloin (**66** and **95**), and microdontin (**100**) and its isomers (**101**, **105**, and **108**).

### 2.2. Identification of Chromones

In total, 36 compounds (**6**–**14**, **18**–**19**, **21**, **23**–**24**, **29**, **36**, **50**–**51**, **54**–**55**, **64**, **67**–**68**, **71**–**72**, **81**–**82**, **90**–**91**, **92**, **94**, **96**, **98**–**99**, and **106**–**107**) from *Aloea barbadenis* Miller were discriminated in KY, which had C-glucosylated 5-methylchromones [38,48] as their main skeleton. These compounds were classified as chromones using diagnostic aglycone ions at *m*/*z* 231 (C_13_H_11_O_4_)/*m*/*z* 233 (C_13_H_13_O_4_) and at *m*/*z* 243 (C_14_H_11_O_4_)/*m*/*z* 245 (C_14_H_13_O_4_)/*m*/*z* 247 (C_14_H_15_O_4_), with detection in negative or positive ion modes.

Compound (**6**) exhibited [M+H]^+^ at *m*/*z* 395.1339 with a molecular formula of C_19_H_23_O_9_; CFIs of *m*/*z* 275.0913 ([M+H-C_4_H_8_O_4_]^+^), *m*/*z* 245.0821 ([275-CH_2_O]^+^), and *m*/*z* 233.0808 ([245-CHO]^+^) were produced in the MS^2^ spectrum through the loss of a glucose unit and two CHO unit molecules. Through a comparison with the literature [48], compound **6** was tentatively identified as aloesin, and two compounds were identified as its isomers (**7** and **8**); compound **14** was identified as 7-O-methylaloesin. Similarly, based on the above main fragments and a comparison to the literature data [6,7,43,49,50,51], compound **10** was putatively identified as 8-C-glucosyl-(R)-aloesol, compound **11** was identified as 8-C-glucosyl-7-O-(S)-methyl aloesol, compound **12** was identified as (2′R)-8-C-glucosyl aloesol, and compound **23** was identified as 8-C-glucosyl-(S)-aloesol. In addition, six compounds were tentatively assumed to be isomers and derivatives of 8-C-glucosyl-aloesol (**9**, **13**, **18**–**19**, **21**, and **24**).

Compound (**36**) exhibited [M+H]^+^ at *m*/*z* 559.181 2 with a molecular formula of C_28_H_31_O_12_, and the MS^2^ spectrum displayed main fragment ions of *m*/*z* 397.1464 ([M+H-C_6_H_10_O_5_]^+^), *m*/*z* 353.1246 ([M+H-C_2_H_4_O-C_9_H_6_O_2_]^+^) *m*/*z* 277.1080 ([353-C_2_H_4_O_2_]^+^), and *m*/*z* 233.0807 ([277-C_2_H_4_O]^+^), indicating the existence of a coumaroyl group and 8-C-glucosyl aloesol group [6,7,43,49,50,51]; hence, compound **36** was putatively identified as coumaroyl-O-8-C-glucosyl aloesol, which was identified for the first time in *Aloea barbadenis* Miller.

Compound (**67**) had [M+H]^+^ at *m*/*z* 557.2021 with a molecular formula of C_29_H_33_O_11_, and the MS^2^ spectrum revealed a main fragment ion of *m*/*z* 513.0758 ([M+H-C_2_H_4_O]^+^) and diagnostic ions of *m*/*z* 393.1326 ([513-C_4_H_8_O_4_]^+^) (formed through the loss of glucosyl) and *m*/*z* 247.0995 ([393-C_9_H_6_O_2_]^+^) (formed through the loss of coumaroyl), which indicated the existence of aloesol [6,7,43,49,50,51]. Hence, compound **67** was confirmed to be aloeresin D through a comparison of the retention time and fragmentation pattern with a standard reference. In addition, compound **64** was identified as its isomer, compound **29** was putatively identified as 4′-O-glucosyl-isoaloeresin D, and four compounds, **68**, **91**, **96**, and **98**, were assumed to be aloeresin D derivatives.

**Figure 3:**
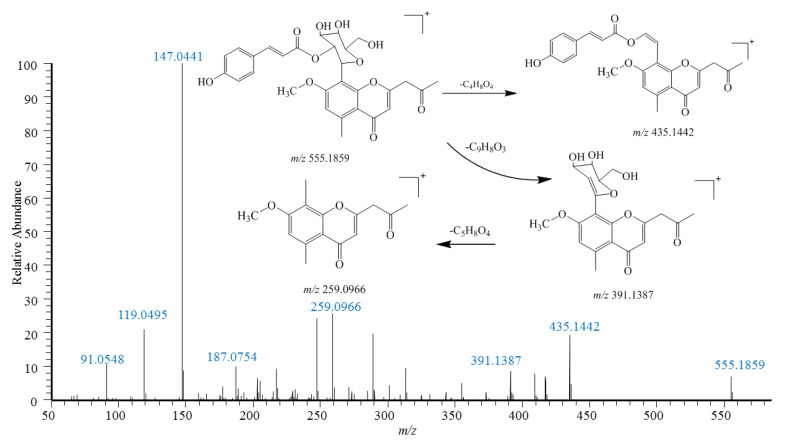
MS/MS spectra and proposed fragmentation pathways of 7-O-methylaloeresin A (**72**) from *Aloea barbadenis* Miller, which was identified in KY.

Compound (**72**) exhibited [M+H]^+^ at *m*/*z* 555.1859 with a molecular formula of C_29_H_29_O_11_. CFIs of *m*/*z* 435.1442 ([M+H-C_4_H_8_O_4_]^+^), *m*/*z* 391.1387 ([M+H-C_9_H_8_O_3_]^+^) and *m*/*z* 259.096 6 ([391-C_5_H_8_O_4_]^+^) were produced in the MS^2^ spectrum (Figure 3); compared with reference compounds, the compound was assumed to be 7-O-methylaloeresin A (**72**), and compound **71** was its isomer.

Based on the CFIs and proposed fragmentation pathways of the above compounds and data base searches (Compound Discoverer/Chem Blink/Chem Spider), seven compounds were identified as chromones [6,7,43,49,50,51]: 2′-O-coumaroyl aloesin (**50** and **51**), rabaichromone (**54** and **55**), aloeresin F (**81**), aloeresin E (**92**), and aloeresin A (**99**). In addition, five compounds (**82**, **90**, **94**, **106**–**107**) were identified as O-glucosylated chromones [49,50,51], which corresponded with the existence of aglycone ions at *m*/*z* 243.1017 (C_15_H_15_O_3_), *m*/*z* 247.0957 (C_14_H_15_O_4_), or *m*/*z* 247.0653 (C_13_H_11_O_5_) in the MS^2^ spectrum.

### 2.3. Identification of Phenylpyrones

A total of 12 compounds from *Aloea barbadenis* Miller were identified in KY, which had six phenylpyrones as their basic structure [38,49,50,51]. During mass spectrometric cleavages, phenylpyrones easily lost their glucose unit to produce aglycone ions, which were obtained as typical fragment ions of *m*/*z* 247 [M-H]^−^ or *m*/*z* 249 [M+H]^+^.

In comparison to the reference substance, compound (**32**) was assumed to be aloenin A, possessing [M-H]^−^ at *m*/*z* 409.1148 with a molecular formula of C_19_H_23_O_10_. In the MS^2^ spectrum, the CFI spectrum at *m*/*z* 247.0615 ([M-H-C_6_H_10_O_5_]^−^) was obtained, which involved the continuous loss of CHO and CO_2_ molecules and produced fragment ions of *m*/*z* 215.0344 and *m*/*z* 203.0716. Three compounds, **16**, **22**, and **33**, were identified as its isomers. For compound **58**, a CFI was obtained at *m*/*z* 249.0757 in the MS^2^ spectrum; through a comparison with the existing literature [49,50,51], it was tentatively identified as aloenin B. In addition, compound **53** was assumed to be aloenin aglycone, and compounds **15** and **17** were identified as 10-O-β-D-glucopyranosyl aloenin and its isomer.

Compound (**43**) showed [M-H]^−^ at *m*/*z* 733.2010 with a molecular formula of C_34_H_37_O_18_, and main fragments were obtained at *m*/*z* 571.1466 ([M-H-C_6_H_10_O_5_]^−^), *m*/*z* 409.1150 ([M-H-2C_6_H_10_O_5_]^−^), and *m*/*z* 247.0613 ([M-H-3C_6_H_10_O_5_]^−^) in the MS^2^ spectrum; through a comparison with aloenin, it was assumed to be aloenin-O-dihexoside, and compound **42** was its isomer. Aloenin-O-dihexoside was identified in *Aloea barbadenis* Miller for the first time.

Compound (**88**) exhibited [M+H]^+^ at *m*/*z* 557.1470 with a molecular formula of C_28_H_29_O_12_. In the MS^2^ spectrum, a CFI at *m*/*z* 249.0758 ([M+H-C_9_H_6_O_2_-C_6_H_8_O_5_]^+^) was observed, formed through the loss of coumaroyl and glucose units. Based on the literature [49,50,51], compound **88** was tentatively identified as aloenin-2′-coumaroyl ester, and compound **89** was its isomer.

### 2.4. Identification of Triterpenes

#### 2.4.1. Cucurbitacin-Type Triterpenes

Six compounds from *Citrullus Colocynthis* (L.) were identified in the KY, all of which belonged to cucurbitacin-type triterpenes. During mass spectrometric cleavages, cucurbitacines easily lose side chains, substituents, and glucose units [8,11].

Compound (**79**) exhibited an adduct ion, [M-H+HCOO]^−^, at *m*/*z* 721.3452 with a molecular formula of C_37_H_53_O_14_ and [M-H]^−^ at *m*/*z* 675.3391 with a molecular formula of C_36_H_51_O_11_. The main fragment at *m*/*z* 513.2853 ([M-H+HCOO-C_6_H_10_O_5_]^−^) was obtained in the MS^2^ spectrum due to the loss of a glucose unit; compared with existing reports [8,52], the compound was tentatively identified as cucurbitacin I-2-O-glucoside. In addition, compound **78** was identified as a cucurbitacin I derivative, and compound **80** was putatively assumed to be cucurbitacin L-2-O-glucoside.

**Figure 4:**
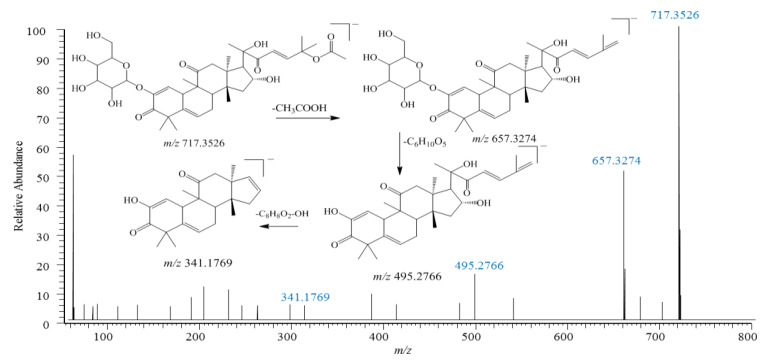
MS/MS spectra and proposed fragmentation pathways of cucurbitacin E-2-O-glucoside (**103**) from *Citrullus Colocynthis* (L.), which was identified in KY.

Cucurbitacin E-2-O-glucoside (**103**) was identified according to reference compounds (Figure 4), producing an adduct ion, [M-H+HCOO]^−^, at *m*/*z* 763.3551 with a molecular formula of C_39_H_55_O_15_ and [M-H]^−^ at *m*/*z* 717.3501 with a molecular formula of C_38_H_53_O_13_. The following fragment ions were also observed in the MS^2^ spectrum: [M-H-CH_3_COO]^−^ at *m*/*z* 657.3280 (formed through the loss of an acetyl group) and [657-C_6_H_10_O_5_]^−^ at *m*/*z* 495.2775 (formed through the loss of a glucose unit). And then, the fragment at *m*/*z* 495.2775 further lost a side chain group (C_8_H_8_O_2_), resulting in *m*/*z* 341.1769. Based on the main fragments and fragmentation rules of the above cucurbitacin compounds, compounds **104** and **113** were tentatively identified as cucurbitacin derivatives.

#### 2.4.2. Tirucallane-Type Triterpenes

A total of nine compounds from *Pistacia lentiscus* L. were distinguished in KY, which were categorized as tirucallane-type triterpenes. During mass spectrometric cleavages, tirucallanes tend to lose their side chain, D-ring, and C-ring groups [35,36,37].

Compound (**114**) had [M+H]^+^ at *m*/*z* 457.3678 with a molecular formula of C_30_H_49_O_3_. In the MS^2^ spectrum, the main fragment was observed at *m*/*z* 457.3678 ([M+H-C_8_H_14_O_2_]^+^), form through the loss of the side chain group. Compound (**123**) exhibited a diagnostic fragment at *m*/*z* 271.1699 ([M-H-C_8_H_12_O_2_-C_3_H_6_-CO]^−^) in the MS^2^ spectrum, which was produced through continuous losses of the side chain, D-ring, and C-ring. Additionally, for compound (**126**), a main fragment was also obtained at *m*/*z* 293.189 2 [M+H-C_7_H_12_O_2_-C_2_H_6_]^+^, formed through the loss of the side chain and D-ring. The main fragments and fragmentation rules of the above compounds were similar to those found in our previous study [35,36,37]; therefore, compounds **114**, **115**, **123**, and **126** were putatively identified as tirucallane-type triterpenes.

(3S,11S)-3-acetoxy-7-hydroxy-11-oxo-tirucalla-8,24 (Z)-dien-26-oic acid (**127**) was identified according to the compounds isolated in-house [35,36,37], producing main fragment ions at *m*/*z* 467.3714 ([M-H-CH_3_COO]^−^) and at *m*/*z* 301.2151 ([467-C_8_H_12_O_2_-C_2_H_4_]^−^) in the MS^2^ spectrum due to the successful loss of acetyl, side chain, and D-ring groups. Based on our previous study, four compounds were tentatively identified as (7R)-7-hydroxy-3,11-dioxo-tirucalla–8,24 (Z)-dien-26-oic acid (**119**), 3,7-dioxo-11-β-hydroxy-tirucalla-8,24 (Z)-dien-26-oic acid (**124**), 3-oxo-tirucalla-5,7,24 (Z)-trien-26-oic acid (**129**), and (3S,9R)-3-acetoxy-6-oxo-tirucalla-7,24 (Z)-dien-26-oic acid (**134**) [35,36,37].

#### 2.4.3. Olean- and Lupane-Type Triterpenes

Ten compounds from *Pistacia lentiscus* L. belonging separately to the olean and lupane types of pentacyclic triterpenes were discriminated in KY.

Compound (**116**) exhibited [M+H]^+^ at *m*/*z* 458.2253 with a molecular formula of C_30_H_50_O_3_. The main fragments at *m*/*z* 441.3716, *m*/*z* 249.1845, and *m*/*z* 201.1645 (determined through RDA cleavages) were also observed in the MS^2^ spectrum; through a comparison with our previous study, the compound was tentatively identified as an olean-type triterpene.

Through a comparison with reference compounds [35,36,37], compound **131** was assumed to be 3,4-secoolean-4(24):18-dien-3,28-dioic acid and belonged to the olean type, with CFIs at *m*/*z* 425.3417, *m*/*z* 407.3307 (formed through the loss of the C-3/C-17 hydroxyl group), and *m*/*z* 235.1689 (formed due to the effect of RDA cleavages on the C-ring) in the MS/MS spectrum. Compound **135** was identified as its isomer. Compounds **136** and **144** were also obtained, exhibiting a fragment ion at *m*/*z* 235.1689 in the MS^2^ spectrum, andwere recognized as olean aldehydes. Furthermore, the MS/MS fragment ions of the two compounds were analyzed and compared to reference compounds of the olean type, and they were tentatively identified as 3,11-dioxo-28-norolean-12-en-17-ol (**139**) and 28-hydroxy-β-amyrone (**140**).

Similarly, through a comparison with reference compounds [35,36,37], compound **143** was recognized as lupane aldehyde; CFIs at *m*/*z* 247.1697, *m*/*z* 213.1641 (CD-ring ions), and *m*/*z* 177.1630 (AB-ring ions) were observed in the MS^2^ spectrum due to RDA cleavages [47]. Furthermore, compounds **141** and **142** also contained lupane-type CFIs (at *m*/*z* 245.1895 and *m*/*z* 177.1632), so they were identified as lupane derivatives [53].

### 2.5. Identification of Resin Glycosides

A total of 12 resin glycosides from *Convolvulus scammonia* L. were identified in the KY. According to a previous report about resin glycosides [25], jalapinolic acid (with main fragments at *m*/*z* 271) is the most frequent aglycone in a macrocycilc lippooligosaccharide core, and some small molecules of organic acids [19], such as 3-hydroxy-2-methylbutyric acid (Nia), tiglic acid (Tga), isobutyric acid (Iba), and 2-methylbutyric acid (2-Mba) also have intra-linkages with this macrocycilc structure.

**Figure 5:**
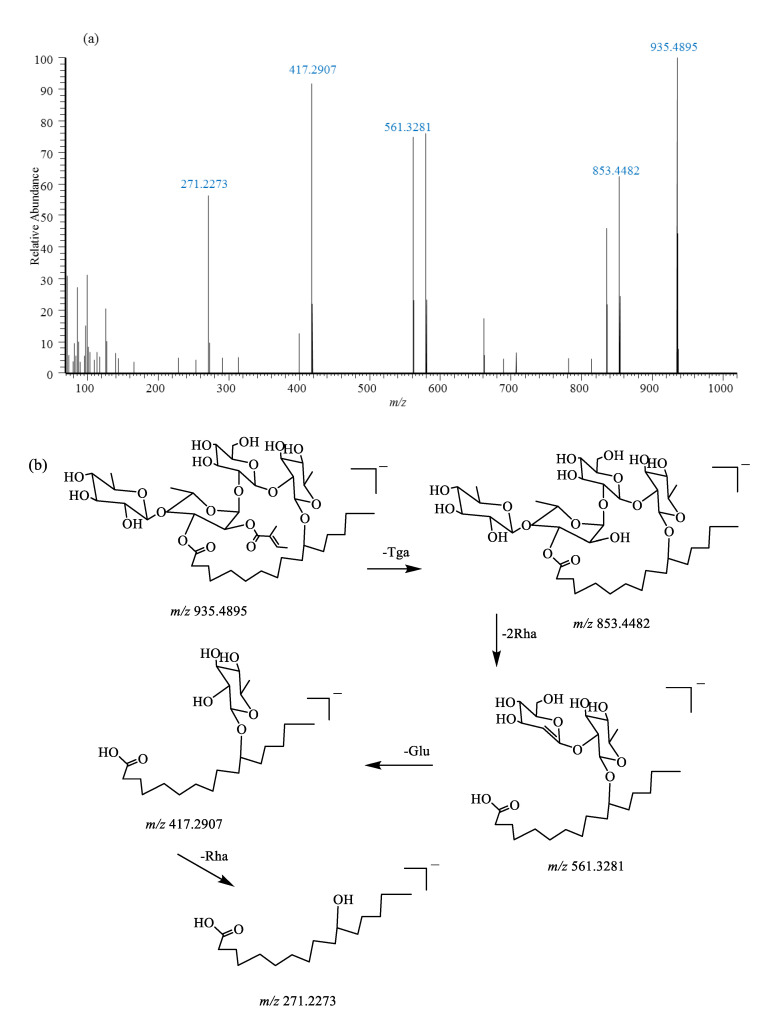
MS/MS spectra (**a**) and proposed fragmentation pathways (**b**) of scammonin VI (**118**) from *Convolvulus scammonia*, which was identified in the KY.

Compound (**118**) exhibited an adduct ion, [M-H+HCOO]^−^, at *m*/*z* 981.4930 with a molecular formula of C_46_H_77_O_22_ and [M-H]^−^ at *m*/*z* 935.4893 with a molecular formula of C_45_H_75_O_20_. Main fragments at *m*/*z* 853.4482 ([M-H-C_5_H_6_O]^−^) and *m*/*z* 835.4392 ([853-H_2_O]^−^) were observed in the MS^2^ spectrum due to the loss of the tiglic acid group and H_2_O molecules. In addition, a fragment at *m*/*z* 853.4482 continuously lost two rhamnosyl (rha), one glucosyl (glu), and one rhamnosyl groups to separately produce fragment ions at *m*/*z* 561.3281, *m*/*z* 417.2907, and *m*/*z* 271.2273 (Figure 5), indicating the existence of a macrocycilc lippooligosaccharide core (jalapinolic acid + rha + rha + glu + rha + tga), which coincided with the characteristics of scammonin VI previously reported [23].

Based on the fragmentation rules and main fragments of scammonin VI, through comparisons with our previous study and the existing literature [22,23,24,25,54,55], resin glycosides and resin glycosidic acids were tentatively identified in KY, including scammonic acid A + Nia + Tga (**117**), scammonin VIII (**120**), scammonin II (**121**), scammonin II + Nia +Iba (**122**), scammonin VI + Nia (**125**), orizaben III (**128** and **130**), scammonin II (**132**), orizaben XIII (**133**), and orizaben IX (**137** and **138**).

### 2.6. Identification of Phenolic Acids

A total of 10 compounds (**3**–**5**, **20**, **35**, **59**, **62**, **75**–**76**, and **97**) from *Artemisia absinthium* L. and *Convolvulus scammonia* L. were distinguished in the KY.

5-O-caffeoylquinic acid (**4**) was identified according to a reference compound and displayed [M-H]^−^ at *m*/*z* 353.0888 with a molecular formula of C_16_H_17_O_9_, with CFIs mainly obtained at *m*/*z* 191.0563 ([M-H-C_9_H_6_O_3_]^−^), *m*/*z* 179.0349 ([M-H-C_7_H_9_O_5_]^−^), and *m*/*z* 173.0349 ([191-H_2_O]^−^) in the MS^2^ spectrum, indicating the existence of caffeoyl and quinic acid groups. Based on the CFIs and reference compounds, the presence of quinic acid (**3**), 3-O-caffeoylquinic acid (**5**), caffeoylquinic acid derivatives (**20**), 3,5-O-di-caffeoylquinic acid (**59**), and 4,5-O-di-caffeoylquinic acid (**62**) was confirmed.

Compound (**35**) had [M-H]^−^ at *m*/*z* 533.1321 with a molecular formula of C_25_H_25_O_13_. In the MS^2^ spectrum, a quinic acid fragment of *m*/*z* 191.0563 ([M-H-C_9_H_6_O_3_-C_9_H_6_O_3_-H_2_O]^−^) was observed as a base peak. It was putatively assumed to be a type of di-O-caffeoylquinic acid [56].

Compound (**75**) displayed [M-H]^−^ at *m*/*z* 513.1321 with a molecular formula of C_26_H_25_O_11_ and showed CFIs of feruloyl quinic acid, with *m*/*z* 367.1036 ([M-H-C_9_H_6_O_2_]^−^) (formed through the loss of a coumaroyl moiety) in the MS/MS spectrum. So, it was putatively identified as feruloyl-O-coumaroyl quinic acid [56], and compound **76** was its isomer.

Compound (**97**) displayed [M-H]^−^ at *m*/*z* 543.1513 with a molecular formula of C_27_H_27_O_12_. The main fragment (a cinnamoyl moiety) was obtained at *m*/*z* 381.1219 ([M-H-C_9_H_6_O_2_]^−^); through a comparison with the literature, it was tentatively assumed to be cinnamoyl-O-coumaroyl quinic acid.

### 2.7. Identification of Flavonoids

A total of four compounds (**44**, **47**, **56**, and **74**) from *Artemisia absinthium* L., *Citrullus Colocynthis* (L.), and *Aloea barbadenis* Miller were found in the KY.

Compound (**44**) had [M-H]^−^ at *m*/*z* 431.0996 with a molecular formula of C_21_H_19_O_10_ and showed fragment ions at *m*/*z* 311.0508 ([M-H-C_4_H_8_O_4_]^−^) and *m*/*z* 283.0612 ([311-CO]^−^) in the MS^2^ spectrum, which were produced through the loss of a glucose unit and CO molecules; through a comparison with a reference compound, it was confirmed to be isovitexin [57].

Compound (**47**) exhibited [M+H]^+^ at *m*/*z* 463.1234 with a molecular formula of C_22_H_23_O_11_ and had fragment ions at *m*/*z* 301.0706 ([M-H-C_6_H_10_O_5_]^−^), *m*/*z* 283.0600 ([301-H_2_O]^−^) (hispidulin aglycone ion), and *m*/*z* 255.0652 ([283-CO]^−^), which were obtained due to loss of glucose unit, H_2_O, and CO molecules. Based on the literature [58,59,60], compound (**47**) was tentatively assumed to be hispidulin-O-glucoside. In addition, for compound (**56**), an aglycone ion was obtained at *m*/*z* 301.0498 ([M-H-C_6_H_10_O_5_]^−^) in the MS^2^ spectrum; through a comparison with a reference, it was confirmed to be isoquercitrin. For compound (**74)**, in the MS^2^ spectrum, the main fragment ion at *m*/*z* 271.0599 ([M-H-C_6_H_10_O_5_]^−^) was found to be the base peak, formed through the loss of the glucose unit; through a comparison with the literature [58,59,60], the compound was tentatively identified as apigenin-O-hexoside.

### 2.8. Identification of Lignanes

In total, two compounds (**111** and **112**) from *Artemisia absinthium* L. were distinguished in the KY.

Compound (**111**) exhibited [M+H]^+^ at *m*/*z* 446.1927 with a molecular formula of C_24_H_30_O_8_; it coincided with the characteristics of diayangambin, based on our previous study [27], and was a 7-O-9′ and 7′-O-9 parallelism link tetrahydrofuran lignan. In the MS^2^ spectrum, a fragment at *m*/*z* 415.1757 ([M+H-OCH_3_]^+^) was observed, produced through the loss of the methoxyl group, and fragment ions at *m*/*z* 250.1197 and *m*/*z* 181.0858 (as a base peak) were produced due to the cleavage of the 7′-O-9 position. In addition, compound (**112**) was also identified as its isomer.

### 2.9. Identification of Others

Compound (**1**) had [M-H]^−^ at *m*/*z* 341.1094 with a molecular formula of C_12_H_21_O_11_ and showed a main fragment at *m*/*z* 179.0536 ([M-H-C_6_H_10_O_5_]^−^) (formed through the loss of the glucose unit) in the MS^2^ spectrum. Through a comparison with the literature, it was putatively identified as maltose [61]. In addition, compound (**2**) was tentatively identified as maltotriose [62]; it showed [M+Na]^+^ at *m*/*z* 527.1589 with a molecular formula of C_18_H_32_O_16_Na and displayed fragments at *m*/*z* 365.1052 ([M+Na-C_6_H_10_O_5_]^+^) and *m*/*z* 203.0524 ([M+Na-2C_6_H_10_O_5_]^+^) in the MS/MS spectrum.

Compound (**110**) displayed [M+H]^+^ at *m*/*z* 359.1115 with a molecular formula of C_19_H_19_O_7_. In the MS^2^ spectrum, a base peak fragment was produced at *m*/*z* 341.1015 ([M+H-H_2_O]^+^), and serious fragmentation occurred at *m*/*z* 299.0911 ([341-C_2_H_2_O]^+^), *m*/*z* 281.0802 ([M+H-2H_2_O-C_2_H_2_O]^+^), and *m*/*z* 271.0967 ([281-CO]^+^), indicating the loss of H_2_O, C_2_H_2_O, and CO groups. Through a comparison with previous studies [63], compound (**110**) was tentatively identified as aloe dihydroisocoumarin A. Compound (**39**) exhibited [M+H]^+^ at *m*/*z* 521.1655 with a molecular formula of C_25_H_29_O_12_ and showed fragments of *m*/*z* 359.1115 (produced through the loss of the glucose unit), *m*/*z* 341.1015, and *m*/*z* 299.0911 in the MS^2^ spectrum, which was similar to compound (**110**) [63]. Therefore, it was assumed to be aloe dihydroisocoumarin A-O-hexoside, and compound (**73**) was its isomer, which was identified in *Aloea barbadenis* Miller for the first time.

## 3. Materials and Methods

### 3.1. Chemicals and Reagents

LC-MS-grade acetonitrile, methanol, and formic acid were purchased from Fisher Scientific (Fair Lawn, NJ, USA). Deionization water was obtained from Watson’s (Xinjiang, China). The other reagents were of analytical grade and bought from the Tianjin Chemical Reagent Corp. (Tianjin, China).

Reference standards: Aloesin, aloin A, aloin B, aloenin A, 7-O-methylaloeresin A, aloeresin D and aloe emodin (purity ≥ 98%) were purchased from Shanghai Pure one Bio-Technology Co., Ltd. (Shanghai, China). The certified reference materials (CRMs) for 3-O-caffeoylquinic acid, 5-O-caffeoylquinic acid, 3,5-O-di-caffeoylquinic acid, and 4,5-O-di–caffeoylquinic acid (purity ≥ 98%) were prepared by the Xinjiang Technical Institute of physics and chemistry, Chinese Academy of Science (Urumqi, China). The standards for isovitexin, cucurbitacin E-2-O-glucoside, and cucurbitacin I (purity ≥ 98%) were bought from Sichuan Heng Cheng Zhi Yuan Bio-Technology Co., Ltd. (Sichuan, China).

### 3.2. Materials

Kukeya tablets (batch no: Y201205) were produced by the Xinjiang Yin Duo Lan Pharmaceutical Limited Liability Company (Urumqi, China). They were composed of five herbal ingredients: 135 g of *Aloea barbadenis* Miller, 135 g of *Artemisia absinthium* (L.), 135 g of *Pistacia lentiscus* (L.), 50 g of *Convolvulus scammonia* L., and 50 g of *Citrullus colocynthis* (L.) Schard.

### 3.3. Sample Preparation

A total of 0.5 g KY powder was precisely weighed, added to 100 mL of a solution with 70% methanol, and then extracted ultrasonically (35 kHz, 500 W) for 30 min. The sample solution was filtered through a 0.22 μm microporous membrane before analysis. The processes for the screening and optimization of the sample solution are provided in the Supplementary Materials.

### 3.4. UHPLC and MS Conditions

The chromatographic separation equipment included an Ultimate 3000 UHPLC^TM^ system (Thermo Fisher Co., Beremin, Germany), equipped with photo-diode array (PDA) detection. The chromatography separation was performed using a Waters Acquity BEH Shield C_18_ column (2.1 × 100 mm, 1.7 μm, Waters, Ireland). The mobile phases were 0.1% aqueous formic acid (A) and acetonitrile (B), the flow rate was 0.2 mL/min, the column temperature was 35 °C, and the injection volume was 2 μL. The optimum gradient elution program was set as follows: 0~5 min, 5~15% B; 5~14 min, 15% B; 14~15 min, 15~17% B; 15~34 min, 17% B; 34~35 min, 17~21% B; 35~40 min, 21% B; 40~44.5 min, 21~28% B; 44.5~50 min, 28% B; 50~66 min, 28~60% B; 66~70 min, 60~95% B; and 70~79 min, 95% B.

High-resolution MS data were recorded using a Q-Exactive^TM^ hybrid Quadrupole-Orbitrap Mass Spectrometer equipped with a heated ESI source (Thermo Fisher Scientific, Bremen, Germany) in both positive and negative ion modes. The HESI parameters were set as follows: a heating temperature of 300 °C; a capillary temperature of 350 °C; a sheath gas pressure of 40 arb, aux gas pressure of 10 arb, and voltage of 3.5 kV in positive modes; and a sheath gas pressure of 38 arb and voltage of −2.8 kV in negative modes. The Orbitrap scan range was set at *m*/*z* 100–1500 with a resolution of 70,000 FWHM in full-scan MS and with a resolution of 17,500 FWHM for the dynamic mass range in MS^2^. MS/MS spectra fragments were obtained at normalized collision energies (NCEs) of 20, 40, and 60. The isolation window was kept at 4.0 *m*/*z*. Xcalibur 4.2 software (Thermo Fisher Scientific, Waltham, MA, USA) was employed to view and process the HRMS full-scan and dd-ms^2^ data.

## 4. Conclusions

The pharmacological substances of KY were systematically studied by UHPLC-Q-Orbitrap-HRMS. The identified compounds essentially covered the major constituents of five medicinal materials in KY, of which 95 compounds belonged to *Aloea barbadenis* Miller, 19 compounds belonged to *Convovulus scammonia* L., 18 compounds belonged to *Pistacia lentiscus* L., 13 compounds belonged to *Artemisia absinthium* L., and 7 compounds belonged to *Citrullus Colocynthis* (L.).

KY has mainly been used to treat migraine in TCM for many decades. The large number of compounds identified in KY have been reported to have various biological activities, and the active ingredients of different classes and sources play synergistic or complementary roles. For example, treatment with aloin A/B is effective in inflammatory processes, bone diseases in cancer, and cardiovascular diseases [64]. Epiyangambin actively inhibits the proliferation of MCF–7 ATCC breast cancer cells [65]. Scammonin I and II were found to be weakly cytotoxic toward human oral epidermoid carcinoma (KB) [66]. Cucurbitacin-type terpene compounds, with cucurbitacin I-2-O-glucoside, cucurbitacin L-2-O-glucoside, and cucurbitacin E-2-O-glucoside as the main cytotoxic chemical markers, efficient inhibit the Cacao–2 and HT29 colon cancer cell lines [67]. The tirucalla-type terpene compound of (3S,9R)-3-acetoxy-6-oxo-tirucalla-7,24 (Z)-dien-26-oic acid have strong cytotoxic effects against RAW264.7 cells [36]. Hence, the results will be a valuable reference for novel clinical applications of the KY tablet.

## Data Availability

All data are contained in the article.

## Supplementary Materials

The following supporting information can be downloaded at: https://www.mdpi.com/article/10.3390/ph18040457/s1, Figure S1. Full wavelength scanning for KY 70% methanol extracts; Figure S2. Full wavelength scanning for KY 100% methanol extracts; Figure S3. Full wavelength scanning for KY water extracts; Figure S4. Different chromatographic columns (for Cortecs C18) λ=245 nm; Figure S5. Different chromatographic columns (for BEH Shield C18) λ=245 nm; Figure S6. Different chromatographic columns (for BEH C18) λ=245 nm; Figure S7. Comparison with KY different extracts under the optimized chromatographic condition; Table S1. Identification and classification of chemical constituents in Kukeya tablet under positive and negative ion modes.
