# Supplementary material for: A Systematic Profiling of the Components of Kukeya Tablets, a Traditional Ethnic Medicine Prescription, by Ultra-High-Performance Liquid Chromatography–Quadrupole/Orbitrap High-Resolution Mass Spectrometry
Source: Pharmaceuticals (Basel). 2025 Mar 24;18(4):457. doi: 10.3390/ph18040457 (PMC12030101; doi:10.3390/ph18040457)
Supplement: Supplementary file 1 [file pharmaceuticals-18-00457-s001.zip › pharmaceuticals-3508029-supplementary.pdf]

Supplementary materials for screening of sample solution:

1. Selection of Extraction Solvent and Detection Wavelength for KY

Three portions of sample powder (batch number: Y201205), each weighing 0.5 g, were accurately weighed and separately treated with 70% methanol, 100% methanol, and water. The samples were prepared according to the method described in Section 3.3. The analysis was performed using Waters ACQUITY UPLC BEH Shield C_{18} column (2.1 mm × 100 mm, 1.7 μm). The mobile phase consisted of 0.1% formic acid aqueous solution (A) and acetonitrile (B) with the following gradient elution program: 0-15 min, 5%-50% B; 15-20 min, 50%-95% B; 20-25 min, 95% B. The column temperature was set at 30°C, with a flow rate of 0.2 mL/min and an injection volume of 2 μL. PDA full-wavelength analysis was conducted to observe the UV absorption characteristics of the extracts within the range of 190-400 nm. The UV absorption characteristics of the extracts obtained using the three solvents are shown in Figures S1-S3.

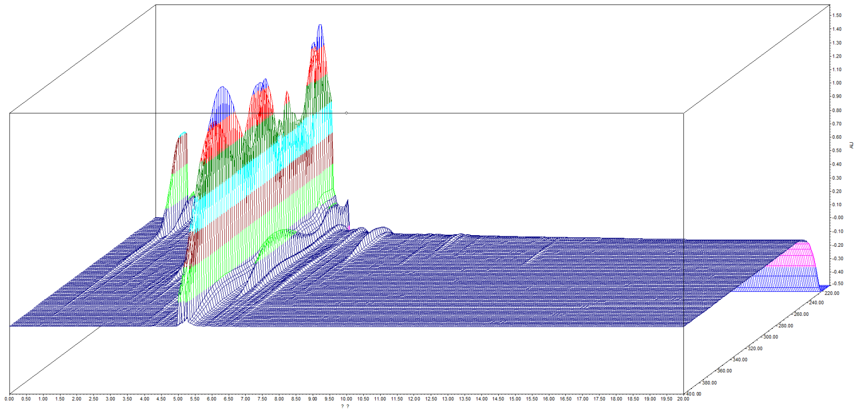

Figure S1. Full wavelength scanning for KY 70% methanol extracts

Note- X: Time (min); Y: UV absorption for chromatographic peaks (AU); Z: Wavelength (nm)

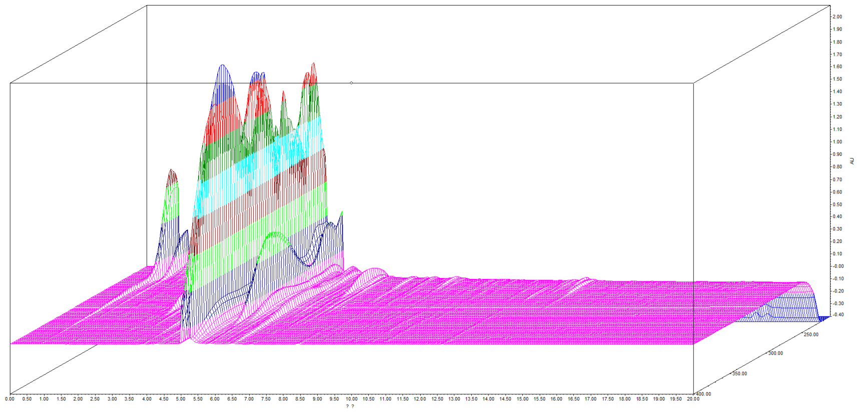

Figure S2. Full wavelength scanning for KY 100% methanol extracts

Note- X: Time (min); Y: UV absorption for chromatographic peaks (AU); Z: Wavelength (nm)

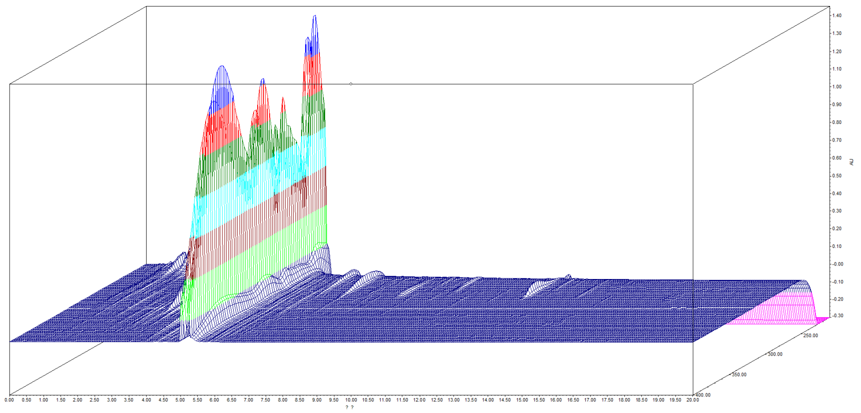

Figure S3. Full wavelength scanning for KY water extracts

Note- X: Time (min); Y: UV absorption for chromatographic peaks (AU); Z: Wavelength (nm)

From the UPLC-PDA full-wavelength scanning profiles of the three sample extracts, it can be observed that the UV absorption of the chromatographic peaks is strongest in the wavelength range of 250-350 nm, indicating a high peak enrichment band. In terms of the number of chromatographic peaks, both major and minor peaks are most abundant in the extract obtained with 70% methanol. Therefore, the sample extract prepared with 70% methanol was selected for further analysis. Considering the full-wavelength UV absorption characteristics and the main components of the five medicinal materials in the formulation, the optimal UV wavelengths were chosen as 245 nm, 280 nm, and 320 nm.

2. Selection of different chromatographic column for KY

Three different chromatographic columns were selected for the analysis: Waters ACQUITY UPLC BEH Shield C_{18} (2.1 mm × 100 mm, 1.7 µm), Waters ACQUITY UPLC BEH C_{18} (2.1 mm × 100 mm, 1.7 µm), and Waters CORTECS C_{18} (2.1 mm × 75 mm, 2.7 µm).

The sample preparation and chromatographic conditions were the same as described above. The results of different chromatographic column of 70% methanol extraction of KY are shown in Figures S4-S6.

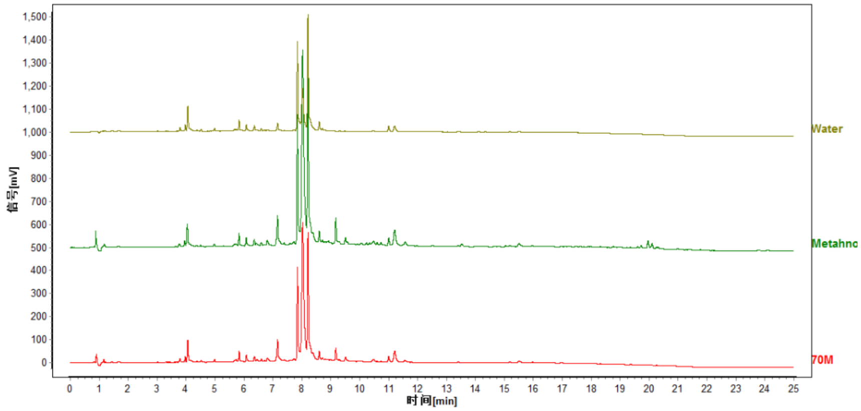

Figure S4. Different chromatographic columns (for Cortecs C_{18}) λ=245 nm

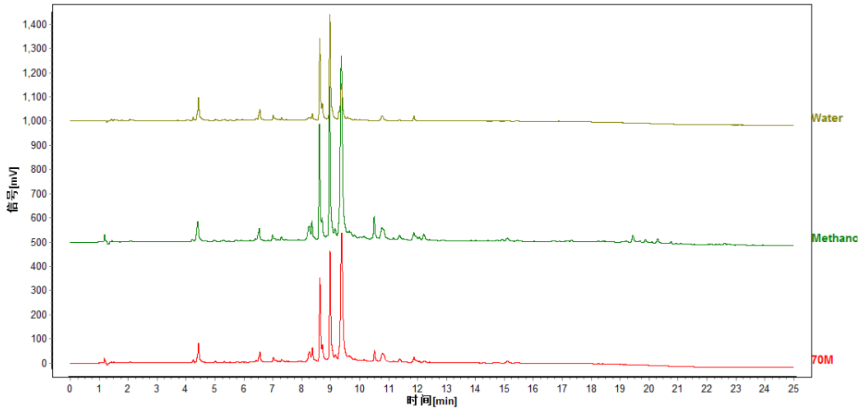

Figure S5. Different chromatographic columns (for BEH Shield C_{18}) λ=245 nm

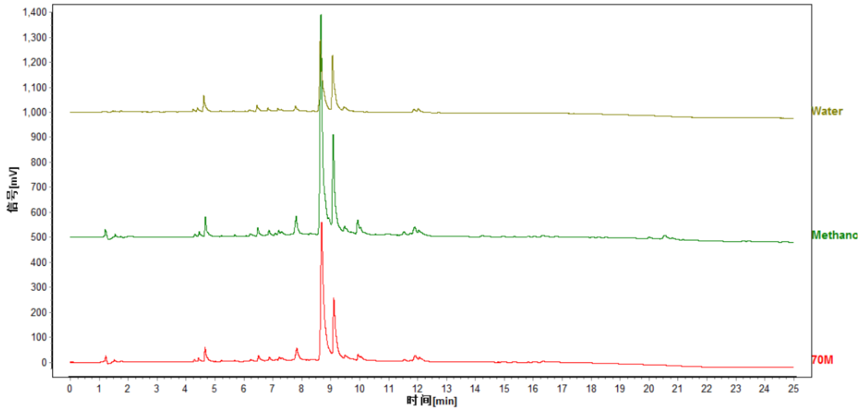

Figure S6. Different chromatographic columns (for BEH C_{18}) λ=245 nm

After comparing the separation efficiency of different chromatographic columns,

it was found that the sample extract is most suitable for the Waters ACQUITY UPLC BEH Shield C_{18} column. This column demonstrated excellent separation efficiency with a large number of well-resolved peaks, effectively separating the complex components in the sample.

3. Optimizing for chromatographic condition

The Waters ACQUITY UPLC BEH Shield C_{18} column (2.1 mm × 100 mm, 1.7 μm) was employed for the analysis, with sample preparation conducted as previously described. Under the optimized chromatographic conditions, the separation efficiency of the chromatographic peaks was compared between the extracts obtained using 70% methanol and pure methanol.

The optimized chromatographic conditions: the mobile phase consisted of 0.1% formic acid aqueous solution (A) and acetonitrile (B) with the following gradient elution program: 0-5 min,5%-15% B; 5-14 min,15% B; 14-15 min, 15%-17% B; 15-34 min, 17% B; 34-35 min, 17%-21% B; 35-40 min, 21% B; 40-44.5 min, 21%-28% B; 44.5-50 min, 28% B; 50-66 min, 28%-60% B; 66-70 min, 60%-95% B; 70-79 min, 95% B. The column temperature was set at 30°C with a flow rate of 0.2 mL/min and an injection volume of 2 μL.

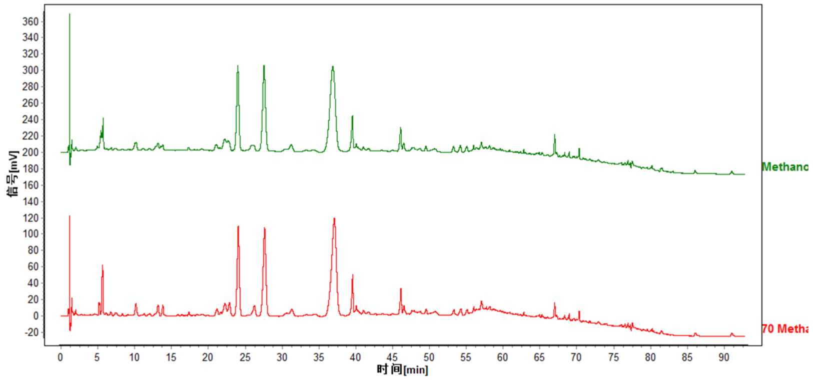

Figure S7. Comparison with KY different extracts under the optimized chromatographic condition

As depicted in Figure S7, under the optimized chromatographic conditions, the extract prepared with 70% methanol demonstrated a greater number of chromatographic peaks and superior separation efficiency. Consequently, the 70% methanol extract of KY was chosen for the identification of its chemical constituents

via LC-HRMS.

Table S1. Identification and classification of chemical constituents in Kukeya tablet under positive and negative ion modes.

N	RT. (mi	Molecular formula	Err pp	Molecular ion	MS/MS fragmentation (relative abundance, %)	Identified compounds	Classification	Sources
**1**	1.28	$C_{12}H_{21}O_{11}$	4.5	341.1094[1)]	179.0562 (25), 119.0349 (30), 89.0244 (80)	Maltose	J	a ~ e
**2**	1.42	$C_{18}H_{32}O_{16}$	1.2	527.1589[4)]	365.1052 (60), 203.0529 (40)	Maltotriose	J	a ~ e
**3**	1.63	$C_7H_{12}O_6$	-	191.0562[1)]	127.0399 (15), 93.0347 (10), 85.00295 (60)	Quinic acid	E	a, b, d
**4**	5.35	$C_{16}H_{17}O_9$	4.8	353.0888[1)]	191.0563 (100), 179.0349 (40), 173.0455 (60), 135.0454 (50)	5-O-caffeoylquinic acid	E	a, b, d
**5**	5.47	$C_{16}H_{17}O_9$	4.8	353.0885[1)]	191.0561 (80), 179.0350 (50), 135.0453 (40)	3-O-caffeoylquinic acid	E	a, b, d
**6**	5.99	$C_{19}H_{23}O_9$	0.0	395.1339[3)]	275.0913 (90), 245.0808 (60), 233.0808 (100), 203.0702 (60)	Aloesin	A	a
**7**	6.07	$C_{19}H_{21}O_9$	2.8	393.1193[1)]	273.0768 (100), 245.0820 (70), 231.0662 (30), 203.0713 (40)	Aloesin isomer	A	a
**8**	6.24	$C_{19}H_{21}O_9$	4.5	393.1198[1)]	273.0769 (60), 245.0822 (30), 231.0664 (60), 203.0715 (100)	Aloesin isomer	A	a
**9**	6.25	$C_{17}H_{21}O_8$	-	353.1229[3)]	233.0807 (100), 203.0703 (85), 176.0834 (25)	8-C-glucosyl-aloesolder	A	a
**1**	6.26	$C_{19}H_{25}O$	-	397.149	277.1017 (30), 243.1015 (30),	8-C-glucosyl-(R)-	A	a

		9		3[3)]	233.0807 (100), 203.0702 (80)	aloesol		
**1**	6.40	$C_{21}H_{27}O_{11}$	3.2	455.1563[2)]	365.1248 (100), 275.0930 (20), 243.0664 (100)	8-C-glucosyl-7-O-(S)-methyl-aloesol	A	a
**1**	6.47	$C_{19}H_{24}O_{9}$	1.5	395.1357[1)]	351.1090 (40), 275.0929 (20), 231.0666 (85), 203.0717 (100)	(2’R)-8–C-glucosyl-aloesol	A	a
**1**	6.76	$C_{20}H_{27}O_{9}$	-	411.1636 [3)]	367.1385 (100), 277.1069 (20), 247.0963 (100)	8-C-glucosyl-7-O-(S)-methyl-aloesol isomer	A	a

No	RT (m	Molecular formula	Err pp	Molecular ion	MS/MS fragmentation (relative abundance, %)	Identified compounds	Classification	Sources
**14**	6.	$C_{20}H_{23}O_{9}$	4.7	407.1356[1)]	275.0923 (30), 243.0663 (100), 234.0528 (20)	7-O-methylaloesin	A	a
**15**	7.	$C_{26}H_{33}O_{17}$	2.9	617.1730[2)]	409.1147 (70), 247.0613 (100), 203.0715 (30)	10-O-β-D-glucopyranosyl-aloenin	C	a
**16**	7.	$C_{19}H_{23}O_{10}$	-	411.1286[3)]	367.1387 (20), 249.0755 (100), 231.0651 (20)	Aloenin	C	a
**17**	7.	$C_{25}H_{33}O_{15}$	-	573.1815[3)]	411.1282 (20), 249.0755 (100), 207.0659 (25)	10-O-β-D-glucopyranosyl-aloenin isomer	C	a
**18**	7.	$C_{22}H_{27}O_{12}$	4.0	483.1523[2)]	393.1180 (100), 275.0922 (45), 231.0669 (90)	8-C-glucosyl-aloesol	A	a

						der		
**19**	7.	$C_{21}H_{27}O_{10}$	-	439.1599[3)]	395.1335 (10), 275.0910 (20), 245.0800 (20), 233.0808 (30)	8-C-glucosyl-aloesol der	A	a
**20**	7.	$C_{16}H_{15}O_{9}$	0.6	351.0713[1)]	191.0561 (100), 173.0458 (20), 135.0455 (10)	Caffeoyl quinic acid der	E	b
**21**	8.	$C_{21}H_{27}O_{10}$	4.0	439.1617[1)]	275.0910 (25), 259.0966 (90), 233.0806 (20)	8-C-glucosyl-aloesol der	A	a
**22**	9.	$C_{19}H_{21}O_{10}$	3.0	409.1154[1)]	247.0616(100), 203.0817 (20)	Aloenin isomer	C	a
**23**	9.	$C_{19}H_{25}O_{9}$	-	397.1492[3)]	277.1069 (40), 243.1010 (20), 233.0808 (100), 203.0702 (80)	8-C-glucosyl-(S)-aloesol	A	a
**24**	9.	$C_{19}H_{25}O_{9}$	0.1	397.1494[3)]	277.1075 (40), 259.0964 (80), 243.1017 (30), 233.0809 (100), 203.0703 (80)	8-C-glucosyl-(S)-aloesol isomer	A	a
**25**	9.	$C_{21}H_{23}O_{10}$	5.0	435.1341[1)]	270.0532 (100), 253.0510 (40)	10-hydroxyaloin der	B	a
**26**	10	$C_{27}H_{33}O_{14}$	3.5	581.1885[3)]	419.1339 (20), 257.0808 (100), 229.0864 (20)	Aloin-O-hexoside	B	a

No	RT (m	Molecular formula	Err pp	Molecular ion	MS/MS fragmentation (relative abundance %)	Identified compounds	Classification	Sources
**27**	10	$C_{21}H_{22}O_{11}$	-	449.1104[1)]	417.1223 (20), 297.0766 (100)	Aloin der	B	a

**28**	10	$C_{28}H_{33}O_{16}$	4.5	625.1791[2)]	417.1200 (20), 279.0656 (10), 255.0664 (100), 211.0774 (10)	Aloin-O-hexoside isomer	B	a
**29**	10	$C_{35}H_{43}O_{16}$	0.2	719.2548[3)]	557.2091 (25), 513.1747 (20), 393.1319 (25), 217.0864 (40), 147.0441 (100)	4'-O-glucosyl-isoaloeresin D	A	a
**30**	10	$C_{30}H_{39}O_{17}$	4.1	671.2209[1)]	525.1614 (100), 213.0559 (90), 185.0557 (40)	Aloveroside A	J	a
**31**	10	$C_{24}H_{29}O_{13}$	1.2	525.1609[1)]	279.0581 (10), 233.0675 (25), 213.0560 (100)	Aloveroside A der	J	a
**32**	11	$C_{19}H_{21}O_{10}$	4.5	409.1148[1)]	247.0615 (100), 171.0452 (50)	Aloenin A	C	a
**33**	11	$C_{19}H_{23}O_{10}$	-	411.1284[3)]	249.0756 (100), 231.0651 (20), 207.0655 (20)	Aloenin A isomer	C	a
**34**	11	$C_{21}H_{21}O_{10}$	1.4	433.1143[1)]	270.0532 (100), 253.0510 (40)	10-hydroxyaloin B	B	a
**35**	12	$C_{25}H_{25}O_{13}$	-	533.1321[1)]	358.0806 (15), 191.0562 (100)	Di-O-caffeoyl-quinic acids	E	b
**36**	12	$C_{28}H_{31}O_{12}$	0.2	559.1812[3)]	397.1464 (15), 277.1080 (15), 233.0807 (80), 163.0391 (100)	Coumaroyl-O-8-C-glucosyl-aloesol	A	a
**37**	13	$C_{21}H_{21}O_{9}$	-	417.1179[3)]	267.0652 (100), 239.0703 (40), 211.0757 (20)	Aloin isomer	B	a
**38**	13	$C_{21}H_{21}O$	3.8	433.115	270.0532 (100), 253.0507 (20)	10-hydroxyaloin	B	a

		10		3[1)]		A		
**39**	13	$C_{25}H_{29}O_{12}$	0.2	521.1655[3)]	359.1115 (90), 341.1015 (60), 299.0911 (100), 281.0802 (50), 271.0967 (20)	Aloedihydro-isocoumarin A -O-hexoside	J	a
**40**	14	$C_{27}H_{31}O_{14}$	4.9	579.1737[1)]	459.1325 (40), 297.0767 (100), 251.0719 (10)	6'-O-acetyl-aloin A der	B	a

No	RT (m	Molecular formula	Err pp	Molecular ion	MS/MS fragmentation (relative abundance, %)	Identified compounds	Classification	Sources
**41**	14	$C_{28}H_{33}O_{16}$	3.4	625.1785[2)]	459.1303 (100), 279.0653 (20), 251.0719 (35)	6'-O-acetyl-aloin A der	B	a
**42**	15	$C_{34}H_{39}O_{18}$	0.2	735.2133[3)]	411.1287 (20), 249.0756 (70), 163.0390 (100)	Aloenin-O-dihexoside	C	a
**43**	15	$C_{34}H_{37}O_{18}$	0.1	733.2010[1)]	571.1466 (45), 409.1150 (30), 247.0613 (100)	Aloenin-O-dihexoside	C	a
**44**	16	$C_{21}H_{19}O_{10}$	3.0	431.0996[1)]	341.0667(50), 311.0558 (100), 283.0612 (60)	Isovitexin	D	a, c
**45**	16	$C_{21}H_{22}O_{10}$	3.9	433.1153[1)]	313.0719 (100), 270.0536 (30), 269.0741 (40)	10-hydroxyaloin isomer	B	a
**46**	17	$C_{27}H_{31}O_{14}$	3.3	579.1728[1)]	416.1115 (100), 267.0305 (15), 252.0434 (25), 224.0478 (15)	6'-O-acetyl-aloin A der	B	a

**47**	18	$C_{22}H_{23}O_{11}$	-	463.1234[3)]	301.0706 (60), 283.0600 (100), 255.0652 (20)	Hispidulin-O-glycoside	D	b
**48**	18	$C_{23}H_{23}O_{10}$	-	459.1311[1)]	339.0866 (90), 297.0760 (100), 267.0669 (25), 255.0675 (50)	6’-O-acetyl-aloin A or isomer	B	a
**49**	18	$C_{28}H_{33}O_{16}$	-	625.1791[2)]	459.1308 (100), 279.0667 (30), 251.0719 (40)	6’-O-acetyl-aloin A der	B	a
**50**	18	$C_{28}H_{29}O_{11}$	4.4	541.1729[1)]	409.1148 (15), 247.0615 (100)	2’-O-coumaroyl -aloesin	A	a
**51**	18	$C_{28}H_{31}O_{11}$	-	543.1857[3)]	257.0810 (10), 233.0809 (40), 147.0441 (100)	2’-O-coumaroyl -aloesin	A	a
**52**	19	$C_{22}H_{19}O_{12}$	3.0	475.0886[1)]	431.0988 (80), 268.0376 (100)	Hydroxyaloin der	B	a
**53**	19	$C_{13}H_{11}O_{5}$	4.4	247.0612[1)]	203.077 (40), 188.0479 (40), 171.0450 (100)	Aloenin aglycone	C	a
**54**	19	$C_{29}H_{33}O_{12}$	-	573.1962[3)]	349.178 (25), 247.0963 (60), 205.0860 (30), 163.0390 (100)	Rabaichromone	A	a
**55**	19	$C_{29}H_{31}O_{12}$	4.2	571.1834[1)]	527.1562 (50), 243.0664 (15), 179.0351 (15), 161.0245 (100)	Rabaichromone	A	a
**56**	20	$C_{21}H_{21}O_{12}$	0.4	465.1030[3)]	303.0500 (100), 153.0183 (20)	Isoquercitrin	D	b

No	RT	Molecu	Err	Molecul	MS/MS fragmentation	Identified	Classi	Sourc

	(m	la r formula	pp	ar ion	(relative abundance, %)	compound s	f i c a t i o n	e s
**57**	20	$C_{21}H_{21}O_9$	2.7	417.1192[1)]	297.0766 (100), 268.0741 (20), 225.0557 (15),	Aloin B	B	a
**58**	22	$C_{34}H_{39}O_{17}$	0.1	719.2183[3)]	411.1286 (20), 249.0757 (50), 147.0441 (100)	Aloenin B	C	a
**59**	22	$C_{25}H_{23}O_{12}$	0.7	515.1191[1)]	353.0867 (90), 191.0559 (40), 179.0351 (60), 135.0457 (100)	3,5-Di-O-caffeoylquinic acid	E	b, d
**60**	23	$C_{21}H_{23}O_9$	-	419.1324[3)]	257.0810 (20), 239.0704 (100), 211.0755 (70)	Aloin A	B	a
**61**	25	$C_{21}H_{21}O_9$	4.6	417.1199[1)]	297.0768 (100), 268.0738 (20), 225.0555 (15)	Aloin A isomer	B	a
**62**	25	$C_{25}H_{23}O_{12}$	4.2	515.1206[1)]	353.0881 (90), 191.0560 (100), 179.0351 (70), 135.0453 (80)	4,5-Di-O-caffeoyl-quinic acid	E	b, d
**63**	26	$C_{28}H_{33}O_{15}$	1.9	609.1840[2)]	563.1768 (20), 443.1349 (100), 295.0602 (20), 251.0712 (20)	Aloinoside B	B	a
**64**	27	$C_{29}H_{33}O_{11}$	0.6	557.2021[3)]	513.1758 (60), 393.1326 (20), 349.1283 (10), 217.0860 (50), 147.0441 (100)	Aloeresin D isomer	A	a
**65**	27	$C_{28}H_{33}O_{15}$	-	609.1842[1)]	443.1348 (100), 279.0656 (50), 225.0136 (20)	Aloinoside B isomer	B	a

**66**	29	$C_{22}H_{23}O_9$	3.5	431.1352[1)]	311.0925 (80), 267.0674 (15)	Homonataloin	B	a
**67**	30	$C_{29}H_{33}O_{11}$	2.5	557.2021[3)]	513.0758 (15), 393.1326 (20), 247.0995 (15), 217.0860 (45), 147.0446 (100)	Aloeresin D	A	a
**68**	30	$C_{27}H_{27}O_{10}$	2.4	511.1611[1)]	243.0659 (20), 145.0296 (100)	Aloeresin D der	A	a
**69**	32	$C_{24}H_{25}O_{11}$	0.2	489.1417[1)]	297.0770 (100), 268.0739 (15)	Aloin der	B	a
**70**	32	$C_{21}H_{19}O_9$	4.3	415.1042[1)]	295.0610 (100), 266.0583 (20), 249.0565 (20)	Aloe-emodin-11-O-rhamnoside	B	a

No	RT (m	Molecular formula	Err pp	Molecular ion	MS/MS fragmentation (relative abundance, %)	Identified compounds	Classification	Sources
**71**	33	$C_{29}H_{29}O_{11}$	3.2	553.1722[1)]	407.1350 (100), 275.0923 (15), 243.0662 (35), 145.0296 (100)	7-O-methyl-aloeresin A	A	a
**72**	33	$C_{29}H_{31}O_{11}$	-	555.1859[3)]	435.1442 (20), 391.1387 (20), 259.0966 (35), 247.0966 (40), 217.0862 (15), 147.0441 (100)	7-O-methyl-aloeresin A	A	a
**73**	34	$C_{25}H_{29}O_{12}$	0.6	521.1657[3)]	359.1125 (80), 341.1020 (50), 299.0914 (100), 281.0808 (40), 271.1961 (30), 253.0849 (15)	Aloe dihydro-iso-coumarin A-O-	J	a

						hexoside		
**74**	36	$C_{21}H_{21}O_{10}$	0.1	433.1133[3)]	271.0599 (100), 253.0499 (20), 227.0704 (20)	Apigenin-O-hexoside	D	a
**75**	36	$C_{26}H_{25}O_{11}$	4.3	513.1414[1)]	367.1036 (40), 349.0934 (35), 163.0402 (15), 145.0296 (100)	Feruloyl-O-coumaroyl quinic acid	E	a, d
**76**	36	$C_{26}H_{25}O_{11}$	4.3	513.1414[1)]	513.1399 (100), 367.1036 (60), 349.0934 (30), 163.0402 (15), 145.0296 (80)	Feruloyl-O-coumaroyl quinic acid	E	a, d
**77**	38	$C_{24}H_{25}O_{11}$	3.2	489.1417[1)]	297.0769 (20), 279.0660 (100), 251.0716 (90)	Aloin der	B	a
**78**	38	$C_{30}H_{41}O_{6}$	0.7	497.0902[3)]	479.2783 (40), 437.2682 (15), 385.2007 (45), 297.1852 (25), 123.0806 (60), 111.0809 (100)	Cucurbitacin I-H_2O	F[1]	c
**79**	38	$C_{37}H_{53}O_{14}$	3.0	721.3452[2)]	675.3391 (100), 513.2853 (20), 383.1864 (15)	Cucurbitacin I-2-O-glucoside	F[1]	c
**80**	39	$C_{37}H_{55}O_{14}$	-	723.3609[2)]	677.3526 (100), 497.2913 (80)	Cucurbitacin L-2-O-glucoside	F[1]	c
**81**	40	$C_{28}H_{29}O_{10}$	-	525.1749[3)]	379.1400 (20), 283.0968 (20), 259.0967 (60), 229.0860 (20), 147.0441 (100)	Aloeresin F	A	a
N	RT. (mi	Molecular formula	Err pp	Molecular ion	MS/MS fragmentation (relative abundance, %)	Identified compounds	Classification	Sources

**8**	41.7	$C_{32}H_{35}O_{13}$	3.1	627.2092[1)]	583.1832 (30), 537.1771 (10), 243.0665 (10), 163.0440 (20), 145.0295 (100)	(E)-2-acetonyl-8-(2''-O-cinnamoyl)–β-D-glucopyranosyl-7-methoxy-5-methylchromone-O-hexoside	A	a
**8**	43.2	$C_{23}H_{23}O_{10}$	3.2	459.1317[1)]	297.0766 (100) ,268.0743 (20), 225.0559 (10)	6'-O-acetyl-aloin A or isomer	B	a
**8**	43.3	$C_{30}H_{21}O_{11}$	0.4	557.1081[1)]	459.1327 (15), 297.0768 (50), 253.0540 (10)	6'-O-acetyl-aloin A der	B	a
**8**	43.9	$C_{26}H_{29}O_{12}$	4.5	533.1678[1)]	443.1357 (100), 279.0654 (20), 251.0711 (30)	Aloinoside B der	B	a
**8**	44.0	$C_{30}H_{21}O_{8}$	0.5	389.1233[3)]	257.0812 (25), 239.0703 (100), 211.0742 (10)	Aloin der	B	a
**8**	44.1	$C_{26}H_{29}O_{12}$	2.5	533.1667[1)]	443.1353 (100), 279.0658 (20), 251.0716 (20)	Aloinoside B der	B	a
**8**	44.6	$C_{28}H_{29}O_{12}$	-	557.1470[3)]	249.0758(50),147.0441(100)	2'-coumaroyl ester aloenin	C	a
**8**	44.7	$C_{28}H_{27}O_{12}$	-	555.1511[1)]	247.0614(100),171.0453(50)	2'-coumaroyl ester aloenin	C	a
**9**	44.9	$C_{29}H_{31}O_{10}$	0.5	539.1909[3)]	419.1491 (20), 273.1119 (20), 243.1017 (60)	(E)-2-acetonyl-8-(2''-O-cinnamoyl)-β-D-glucopyranosyl-7-methoxy-5-methylchromone	A	a
**9**	46.3	$C_{31}H_{35}O_{12}$	0.8	599.2130[3)]	555.1587 (20), 393.1324 (15), 247.0965 (50), 217.0860 (25)	Aloeresin D der	A	a

N	RT. (mi	Molecular formula	Err pp	Molecular ion	MS/MS fragmentation (relative abundance, %)	Identified compounds	Classification	Sources
**9**	46.9	$C_{29}H_{33}O_{10}$	0.6	541.2072[3)]	497.1816 (40), 377.1386 (20), 247.0092 (35), 217.0860 (45), 131.0493 (100)	Aloeresin E	A	a
**9**	47.1	$C_{30}H_{27}O_{12}$	4.7	579.1506[1)]	459.1089 (40), 297.0765 (20), 255.0662 (40), 161.0244 (100)	6'-O-acetyl-aloin A der	B	a
**9**	47.7	$C_{30}H_{35}O_{11}$	-	571.2167[3)]	527.1910 (25), 407.1487 (20), 247.0957 (15), 217.0859 (35), 161.0597 (100)	(E)-2-((S)-2-hydroxypropyl)-8-(2'-OCH$_3$-cinnamoyl)-β-D-glucopyranosyl-7-methoxy-5−methylchromone	A	a
**9**	48.2	$C_{23}H_{21}O_{11}$	3.1	473.1105[2)]	311.0562 (100), 267.0655 (20), 239.0317 (15)	Homonataloin	B	a
**9**	48.7	$C_{24}H_{31}O_{9}$	0.5	463.1965[3)]	445.1862 (100), 396.1574 (15), 277.1075 (20), 247.0967 (98), 195.0654 (85), 181.0858 (70)	Aloeresin D der	A	a
**9**	49.3	$C_{27}H_{27}O_{12}$	2.9	543.1513[1)]	381.1195 (20), 179.0351 (80), 161.0236 (60), 135.0453 (100)	Caffeoyl-dimethoxy cinnamoyl quinic acid	E	d
**9**	49.5	$C_{24}H_{31}O_{9}$	4.9	463.1964[3)]	445.1857 (85), 277.1075 (20), 247.0973 (100), 217.0503 (30), 195.0653 (80)	Aloeresin D der	A	a
**9**	49.8	$C_{28}H_{29}O_{12}$	1.2	557.1661[3)]	393.1339 (10), 249.0759 (80), 207.0650 (10), 147.0441 (100)	Aloeresin A	A	a

No	RT	Molecula	Err	Molecular	MS/MS fragmentation (relative abundance, %)	Identified compounds	Classif	Source

	(m	r formula	pp	ion			ication	s
**10**	50	$C_{30}H_{29}O_{11}$	-	565.1704[3)]	385.1077 (30), 357.1122 (40), 291.0653 (15), 239.0704 (70), 211.0761 (20), 147.0411 (100)	Microdontin	B	a
**10**	50	$C_{30}H_{27}O_{11}$	3.3	563.1566[1)]	443.1145 (40), 297.0769 (40), 255.0664 (20), 145.0296 (100)	Microdontin isomer	B	a
**10**	50	$C_{36}H_{37}O_{15}$	-	709.2143[1)]	589.1724 (20), 443.1356 (100), 279.0664 (20), 253.0510 (20), 145.0296 (70)	Microdontin-O-hexoside	B	a
**10**	51	$C_{36}H_{55}O_{15}$	2.0	763.3551[2)]	717.3494 (100), 657.3280 (50), 495.2775 (20), 383.1875 (10)	Cucurbitacin E-2-O-glucoside	F[1]	c
**10**	51	$C_{30}H_{41}O_{6}$	-	497.2897[3)]	497.2901 (90), 479.2816 (30), 340.2018 (25), 315.1950 (20), 161.0962 (55), 123.0807 (45), 111.0808 (35)	Deacetylated cucurbitacin E	F[1]	c
**10**	51	$C_{30}H_{27}O_{11}$	3.1	563.1563[1)]	443.1145 (60), 297.0769 (50), 255.0664 (20), 145.0296 (100)	Microdontin isomer	B	a
**10**	53	$C_{29}H_{29}O_{12}$	4.8	569.1681[1)]	391.1030 (20), 247.0615 (100), 215.0357 (20), 171.0453 (40)	(E)-2-((S)-2-hydroxypropyl)-8-(2′-OCH_3-cinnamoyl)-β-D-glucopyranosyl-7-methoxy-5–methyl-chromone	A	a

No	RT (m	Molecular formula	Err pp	Molecular ion	MS/MS fragmentation (relative abundance, %)	Identified compounds	Classification	Sources
**10**	53	$C_{29}H_{31}O_{12}$	0.3	571.1812[3)]	249.0757 (55), 161.0598 (100)	(E)-2-((S)-2-hydroxypropyl)-8-(2'-OCH_3-cinnamoyl)-β-D-glucopyranosyl-7-methoxy-5-methylchromone	A	a
**10**	53	$C_{30}H_{29}O_{11}$	0.5	565.1708[3)]	239.0707 (20), 211.0753 (10), 147.0441 (100)	Microdontin isomer	B	a
**10**	54	$C_{15}H_{19}O_{5}$	3.0	269.0453[1)]	257.0459 (10), 240.0426 (25), 211.0398 (15)	Aloe–emodin	B	a
**11**	55	$C_{19}H_{19}O_{7}$	-	359.1123[3)]	341.1018 (45), 299.0913 (100), 281.0809 (40), 271.095 (20)	Aloedihydro -isocoumarin A	J	a
**11**	56	$C_{24}H_{30}O_{8}$	-	446.1927[3)]	415.1757 (15), 250.1197 (20), 195.0652 (50), 181.0859 (100), 166.0859 (20)	Diayangambin	H	b
**11**	58	$C_{24}H_{30}O_{8}$	-	446.1929[3)]	446.1926 (100), 181.0858 (100), 166.0625 (30)	Epiyangambin	H	b
**11**	58	$C_{30}H_{41}O_{6}$	0.1	497.2899[3)]	479.2775 (40), 439.2481 (80), 421.2377 (40),	Cucurbitacin der	F[1]	c

					347.2009 (20), 161.0964 (20), 111.0808 (42)			
**11**	61	$C_{30}H_{49}O_3$	0.3	457.3678[3)]	439.3629 (30), 421.3513 (15), 315.2374 (15), 249.1848 (20), 217.1587 (15), 203.1798 (10), 189.1640 (30), 121.1013 (100)	Tirucallaene-type terpenic acid	F[2]	e
**11**	61	$C_{30}H_{45}O_6$	4.4	501.3233[1)]	483.3114 (10), 455.3176 (10), 341.2476 (20)	Tirucallaene-type terpenic acid	F[2]	e

No	RT (m	Molecular formula	Err pp	Molecular ion	MS/MS fragmentation (relative abundance, %)	Identified compounds	Classification	Sources
**11**	65	$C_{30}H_{50}O_3$	-	458.2253[3)]	441.3716 (25), 249.1845 (40), 201.1645 (15), 133.1014 (60)	Olean-type triterpenic acid	G[1]	e
**11**	65	$C_{50}H_{85}O_{23}$	2.0	1053.5498[1)]	1009.5244 (40), 953.4977 (15), 579.3401 (20), 417.2862 (45), 271.2275 (40)	Scammonic acid A +Nia +Tga	I	d
**11**	66	$C_{46}H_{77}O_{22}$	2.9	981.4930[2)]	935.4893 (20), 853.4492 (25), 579.3379 (25), 417.2907 (30), 271.2273 (20)	Scammonin Ⅵ	I	d
**11**	66	$C_{30}H_{45}O_5$	1.9	485.3271[3)]	467.3158 (100), 449.3039 (60), 421.3104 (45), 339.2318 (20), 283.1692 (20), 269.1537 (10), 215.1492 (25)	(7R)-7-hydroxy-3,11-dioxo-tirucalla-8,24 (Z)-dien-26-oic acid	F[2]	e
**12**	67	$C_{51}H_{85}O_{24}$	2.5	1081.5457[1]	1035.5338 (70), 835.4350 (40), 561.3275 (100),	Scammonin Ⅷ	I	d

				)	417.2855 (80), 271.2278 (60)			
**12**	68	$C_{46}H_{79}O_{22}$	2.0	983.5082[2)]	937.5031 (100), 853.4490 (20), 835.4544 (60), 579.3361 (100), 417.2869 (65), 271.2278 (50)	Scammonin Ⅱ	I	d
**12**	68	$C_{55}H_{93}O_{25}$	-	1153.6027[2)]	1109.5760 (100), 1065.5504 (30), 935.4830 (15), 853.4499 (25), 579.3366 (20), 417.2866 (70), 271.2282 (50)	Scammonin Ⅱ +Nia+Iba	I	d
**12**	69	$C_{30}H_{41}O_5$	3.1	481.2964[1)]	407.2596 (40), 299.1656 (10), 271.1699 (10)	Tirucallaene-type terpenic acid	F[2]	e
**12**	69	$C_{29}H_{45}O_4$	-	457.3312[3)]	327.2308 (10), 201.1639 (25), 189.1639 (50), 121.1014 (100)	Tirucallaene-type terpenic acid	F[2]	e

N	RT. (mi	Molecular formula	Err pp	Molecular ion	MS/MS fragmentation (relative abundance, %)	Identified compounds	Classification	Sources
**1**	69.6	$C_{51}H_{85}O_{24}$	-	1081.5446[2)]	935.4866 (50), 835.4349 (15), 617.3554 (100), 561.3285 (80), 417.2867 (60), 271.2279 (40)	Scammonin Ⅵ +Nia	I	d
**1**	69.8	$C_{30}H_{43}O_3$	2.9	451.3220[3)]	309.2201 (20), 293.1892 (25), 215.1432 (20), 187.1479 (30)	Tirucallaene terpenic acid	F[2]	e
**1** #	69.9	$C_{32}H_{47}O_6$	2.9	527.3381[1)]	467.3174 (15), 301.2151 (10), 271.1693 (10)	(3S,11S)-3-acetoxy-7-hydroxy-11-oxo-tirucalla-8,24 (Z)-dien-	F[2]	e

						26-oic acid		
**1**	70.3	$C_{56}H_{93}O_{26}$	0.9	1181.5937[2)]	1083.5598 (20), 935.4875 (20), 835.4641 (10), 661.3803 (70), 617.3542 (85), 561.3277 (55), 417.2822 (60), 271.2286 (30)	Orizaben Ⅲ	I	d
**1** #	70.7	$C_{30}H_{45}O_{3}$	-	453.3361[3)]	435.3387 (80), 407.3319 (10), 271.2065 (10), 245.1896 (10), 217.1588 (30), 161.1325 (50), 133.1013 (60)	3-oxo-tirucalla-5,7,24 (Z)−trien-26-oic acid	F[2]	e
**1**	70.9	$C_{56}H_{93}O_{26}$	0.9	1181.5937[2)]	1035.5382 (20), 935.4875 (20), 835.4641 (10), 661.3803 (70), 617.3542 (85), 561.3277 (55), 417.2822 (60), 271.2286(30)	Orizaben Ⅲ	I	d

N	RT. (mi	Molecular formula	Err pp	Molecular ion	MS/MS fragmentation (relative abundance, %)	Identified compounds	Classification	Sources
**1** #	71.3	$C_{30}H_{47}O_{4}$	-	471.3467[3)]	453.3368 (15), 425.3417 (20), 407.3307 (20), 249.1858 (20), 235.1689 (35), 189.1634 (40), 133.1013 (70), 121.1014 (90)	3,4-secoolean-4 (24):18-dien-3,28-dioic acid	G[1]	e
**1**	71.6	$C_{44}H_{89}O_{25}$	-	1065.5511[2)]	1019.5502 (20), 919.4915 (45), 835.4321 (20), 579.3387 (55), 561.3280 (100), 417.2869 (40), 271.2280 (45)	Scammonin Ⅰ	I	d
**1**	71.8	$C_{55}H_{91}O_{25}$	1.9	1151.5865[2)]	1105.5317 (15), 1005.5281 (55), 961.5040 (25), 817.4252 (15),	Orizaben XIII	I	d

					661.3812 (45), 617.3545 (100), 561.3281 (40), 417.2846 (30), 271.2283 (50)			
**1**	71.9	$C_{32}H_{49}O_5$	-	513.3574[3)]	453.3380 (20), 435.3559 (15), 325.2528 (15), 269.1910 (15), 217.1590 (25), 189.1642 (10)	(3S,9R)-3-acetoxy-6-oxo-tirucalla-7,24(Z)-dien-26-oic acid	F[2]	e
**1**	72.3	$C_{30}H_{47}O_4$	0.1	471.3470[3)]	453.3358 (15), 425.3417 (10), 409.3471 (20), 235.1687 (35), 201.1644 (20), 133.1013 (70)	3,4-secoolean-4(24):18-dien-3,28-dioicacid isomer	G[1]	e
**1**	72.4	$C_{30}H_{49}O_4$	2.7	441.3779[3)]	441.3734 (25), 423.3626 (10), 233.1894 (35), 203.1793 (40), 107.0860 (100)	Olean aldehyde	G[1]	e
**1**	72.4	$C_{56}H_{93}O_{25}$	1.5	1165.6018[2)]	1119.6010 (20), 1019.5401 (60), 919.4874 (15), 835.4347 (15), 617.3549 (100), 561.3279 (80), 417.2859 (60), 271.2276 (60)	Orizaben Ⅸ	I	d

N	RT. (mi	Molecular formula	Err pp	Molecular ion	MS/MS fragmentation (relative abundance, %)	Identified compounds	Classification	Sources
**1**	72.5	$C_{56}H_{93}O_{25}$	2.1	1165.6006[2)]	1119.5936 (15), 1019.5441 (60), 975.5163 (20), 661.3817 (40), 617.3543 (100), 561.3278 (50), 417.2863 (60), 271.2296 (60)	Orizaben Ⅸ	I	d
**1**	72.6	$C_{30}H_{49}O_2$	-	441.3727[3)]	423.3642 (60), 405.3542 (15), 233.1910 (25), 201.1636 (25), 175.1484 (30),	3,11-dioxo-28-norolean-12-en-17-	G[1]	e

#					121.1014 (100)	ol		
1 #	72.7	$C_{30}H_{49}O_2$	-	441.3727[3)]	423.3638 (70), 405.3515 (15), 233.1900 (15), 217.1951 (80), 203.1796 (40), 133.1012 (100), 121.1014 (100)	28-hydroxy-β-amyrone	G[1]	e
1	73.0	$C_{29}H_{45}O$	-	409.3449[3)]	245.1895 (20), 189.1639 (45),	Lupelol	G[2]	e
1	73.9	$C_{30}H_{91}O_6$	2.5	907.6833[1)]	453.3378 (100)	Lupane aldehyde dimers	G[2]	e
1	73.9	$C_{30}H_{47}O_3$	-	455.3517[3)]	437.3409 (40), 409.3463 (60), 327.2320 (25), 247.1697 (15), 213.1641 (20), 203.1794 (70), 177.1638 (70), 133.1012 (60)	Lupane aldehyde	G[2]	e
1	74.1	$C_{30}H_{47}O_2$	0.2	439.3571[3)]	421.347 1(15),235.169 6(20), 203.179 4(25),191.1794 (55), 109.1015 (70)	Olean aldehyde	G[1]	e

Note:

Ionization mode: 1) $[M\text{-}H]^-$; 2) $[M\text{-}H+HCOO]^-$; 3) $[M+H]^+$; 4) $[M+Na]^+$.

Marks: "#": compared with reference substance; "##": compared with compounds isolated in-house; "*": first-identified compounds.

Classification: A—chromones; B—anthraquinones and anthrones; C—phenylpyrone; D—flavonoids; E—phenols; F—tetracyclic triterpenes: cucurbitacin[1], tirucallaene[2];

G—pentacyclic triterpenes: olean[1], lupane[2]; H−lignan; I—resin glycosides; J—others.

Sources: a—*Aloe barbadesnis* Miller; b—*Artemisia absinthium* L.; c—*Citrullus colocynthis* (L.) Schrad; d—*Convovulus scammonia* L.; e—*Pistacia lentiscus* L.

Abbreviations: Nia: 3-hydroxy-2-methylbutyric acid; Tga: tiglic acid; Iba: isobutyric acid; 2-Mba: 2-methylbutyric acid.
